# Sesterterpenes and a New Sterol from the Marine Sponge *Phyllospongia foliascens*

**DOI:** 10.3390/molecules15020834

**Published:** 2010-02-05

**Authors:** Hong-Jun Zhang, Yang-Hua Yi, Fan Yang, Wan-Sheng Chen, Hou-Wen Lin

**Affiliations:** 1 Laboratory of Marine Drugs, Department of Pharmacy, Changzheng Hospital, Second Military Medical University, 415 Fengyang Road, Shanghai 200003, China; E-Mails: chinabludo@126.com (H.-J.Z.); bill1985@126.com (F.Y.); chenwansheng@21cn.com (W.-S.C.); 2 Research Center for Marine Drugs, School of Pharmacy, Second Military Medical University, 325 Guohe Road, Shanghai 200433, China; E-Mail: yiyanghua@hotmail.com (Y.-H.Y.); 3 Hospital of PLA Troops 95746, 22 Jichang Road, Qionglai 611531, China

**Keywords:** sesterterpene, sterol, marine sponge, *Phyllospongia foliascens*

## Abstract

A new scalarane sesterterpene, phyllofolactone M (**1**), and a new sterol, (24*E*)-5*α*,6*α*-epoxystigmasta-7,24(28)-dien-3*β*-ol (**3**), together with a known sesterterpene, phyllofolactone B (**2**), were isolated from the South China Sea sponge *Phyllospongia foliascens*. Their structures were elucidated by spectroscopic analysis and comparison with known compounds. In addition, previous NMR data assignments for the known sesterterpene phyllofolactone B (**2**) were revised.

## 1. Introduction

**Figure 1:**
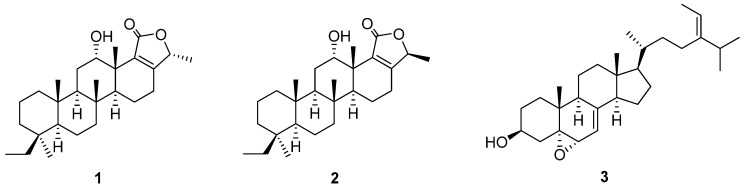
Structures of compounds **1**−**3**.

Scalarane sesterterpenes are typical bioactive secondary metabolites of marine sponges of the genus *Phyllospongia* [1,2]. The sponge *P. foliascens* was known to possess novel sesterterpenes with cytotoxic, antimicrobial, anti-inflammatory and anti-HIV activities, such as foliaspongin [3,4], phyllofoliaspongin [5], phyllactones [6], phyllofenones [7], and phyllofolactones [7−9]. Our previous studies on bioactive constituents of the marine sponge *P. foliascens* collected from the South China Sea have led to the isolation of two new 24-homoscalarane sesterterpenes, phyllofolactone L and phyllofenone D, and a new 20,24-bishomo-25-norscalarane sesterterpene, phyllofenone E [8]. In our continuing investigation on chemical constituents of *P. foliascens*, a new 20,24-bishomoscalarane sesterterpene, phyllofolactone M (**1**), and a new sterol, (24*E*)-5*α*,6*α*-epoxystigmasta-7,24(28)-dien-3*β*-ol (**3**), together with a known compound, phyllofolactone B (**2**), were also obtained from this sponge. Their structures were elucidated by spectroscopic analysis and comparison with known compounds. We herein reported the details of isolation and structure elucidation of compounds **1**−**3** (Figure 1).

## 2. Results and Discussion

The acetone extract of marine sponge *P. foliascens* was subjected to solvent partition, vacuum liquid chromatography (VLC), column chromatography (CC), and RP-HPLC to afford a new 20,24-bishomoscalarane sesterterpene, phyllofolactone M (**1**), and a new sterol, (24*E*)-5*α*,6*α*-epoxystigmasta-7,24(28)-dien-3*β*-ol (**3**), along with a known sesterterpene phyllofolactone B (**2**). Their structures were elucidated by MS, 1D- and 2D-NMR techniques including COSY, HMQC, HMBC and ROESY.

**Figure 2:**
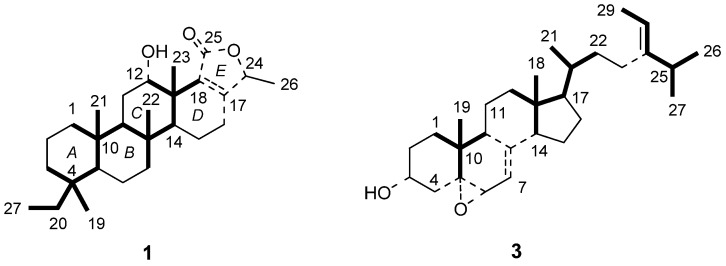
Selected HMBC (bold lines) and COSY (solid lines) correlations of **1** and **3** (Dotted lines indicate bonds without COSY correlations).

Compound **1** was isolated as white powder from CHCl_3_, and its molecular formula was established as C_27_H_42_O_3_ from the HR-TOF-ESI-MS (*m/z* 437.3033, [M+Na]^+^) and ^13^C-NMR data. Seven degrees of unsaturation implied by the molecular formula were ascribed to five rings, one double bond (*δ*_C_ 163.8, 134.2) and one ester carbonyl group (*δ*_C_ 172.0). The ^1^H-NMR spectrum showed six methyl signals at *δ*_H_ 0.74 (3H, t, *J* = 7.4 Hz), 0.79 (3H, s), 0.82 (3H, s), 0.86 (3H, s), 1.19 (3H, d, *J* = 6.7 Hz), and 1.24 (3H, s), two oxymethine signals at *δ*_H_ 5.05 (1H, br. s) and 4.75 (1H, q, *J* = 6.7 Hz), and one hydroxyl signal at *δ*_H_ 5.92 (1H, br. s). The ^13^C-NMR and DEPT spectra exhibited 27 signals, including those of six methyl, nine methylene, five methine and seven quaternary carbons. A typical sesterterpenoid carbons system bearing five methyl groups along rings *A* to *D* could be established by the strong HMBC correlations from the five methyl groups (Me-19, 21, 22, 23, and 27) to the associated carbons, and a 20,24-bishomoscalarane skeleton could be obtained on the basis of further HMBC and COSY correlations (Figure 2). The HMBC correlations from H-16 to C-17 and C-18 confirmed the assignment of ring *D*. The COSY correlations between Me-26 and H-24, and the HMBC correlations form H-24 to C-17, C-18, and C-25 allowed the establishment of the lactone ring *E*. According to the detailed analysis of COSY, HMQC and HMBC spectra, the planar structure of compound **1** was unambiguously determined (Table 1).

**Figure 3:**
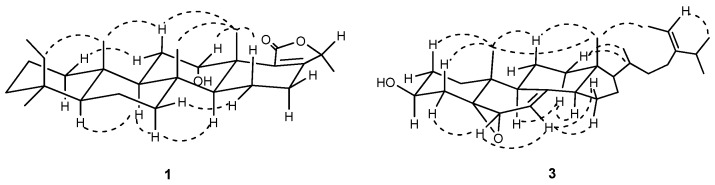
Key ROESY correlations of **1** and **3**.

The ROESY spectrum showed that the rings *A*–*D* were *trans*/*trans*/*trans* fused (Figure 3). The small coupling constants between H-12 (*δ*_H_ 5.05, br. s) and H_2_-11, and the ROESY correlations between H-12 and Me-23 suggested that the HO-12 was *α*-oriented. Because the two protons at C-16 (2H, *δ*_H_ 2.15, m) resonated at the same chemical shit, the orientation of Me-26 could not be deduced from the undistinguished ROESY correlations from Me-26 to H*_α_*-16 and H*_β_*-16, which was determined by following comparison with its epimer, compound **2**.

Compound **2** revealed very similar NMR spectra as compound **1**, and was proven to possess the same molecular formula and planar structure as those of compound **1** by extensive analysis of ESI-MS, 1D- and 2D-NMR spectra. The small coupling constants of H-12 (*δ*_H_ 5.09, br. s) also displayed that the HO-12 of compound **2** was *α*-oriented, suggesting that compounds **1** and **2** were epimers at C-24. Previous NMR and X-ray studies on structurally similar sesterterpenes phyllofolactones and honulactones demonstrated that the C-24 resonates upfield when Me-26 is *β*-oriented compared to the *α*-oriented Me-26, which was diagnostic in confirming the orientation of the Me-26, although the difference was mostly about 0.2−0.3 ppm [10,11]. Accordingly, the structure of compound **2** was determined to be phyllofolactone B with *β*-oriented Me-26 [7], and compound **1** was determined to be a new sesterterpene, 20,24*α*-dimethyl-12*α*-hydroxy-scalaran-25,24-lactone, named phyllofolactone M (Table 2) [8].

Further comparison on ^13^C-NMR, melting point and optical rotation data among compounds **1**, **2** and the reported phyllofolactone B [7] showed that the reported data for phyllofolactone B were almost identical to those of compound **1** (Table 2), indicating that the Me-26 in the literature should be revised to be *α*-oriented, and actually it was compound **1** instead of compound **2** previously obtained.

Compound **3** was isolated as white powder from CHCl_3_, and its molecular formula C_29_H_46_O_2_ was deduced from the TOF-API-MS (*m*/*z* 427, [M+H]^+^) and ^13^C-NMR data. Seven degrees of unsaturation implied by the molecular formula were assigned to five rings and two double bonds (*δ*_C_ 115.7, 117.6, 144.0, 146.9). The ^1^H-NMR spectrum exhibited six methyl groups at *δ*_H_ 0.60 (3H, s), 0.98 (3H, d, *J* = 6.8 Hz), 0.99 (3H, d, *J* = 6.8 Hz), 1.01 (3H, d, *J* = 6.5 Hz), 1.09 (3H, s) and 1.58 (3H, d, *J* = 6.8 Hz), two oxymethine protons at *δ*_H_ 3.63 (1H, br. d, *J* = 4.6 Hz) and 4.08 (1H, tt, *J* = 11.0, 5.0 Hz), and two olefinic protons at *δ*_H_ 5.19 (1H, q, *J* = 6.8 Hz) and 5.36 (1H, m). The ^13^C-NMR and DEPT spectra exhibited 29 signals including those of six methyl, nine methylene, nine methine and five quaternary carbons. The ^1^H- and ^13^C-NMR spectra of compound **3** were characteristic of an oxygenated sterol [12], which was confirmed by extensive 2D-NMR spectroscopic analysis. The strong HMBC correlations from the six methyl groups to associated carbons indicated three typical fragments of steroid corresponding to two angular methyl groups and nearby carbons, and the partial side-chain (Figure 2). A 5*α*,6*α*-epoxy sterol framework could established by the COSY, HMQC and HMBC spectra, which was consistent to the literature [13]. The COSY correlation between H-6 and H-7, together with the HMBC correlations from H-6 to C-5, C-7, C-8 and C-10, and from H-7 to C-5, C-9 and C-14 confirmed the assignment of double bond at C-7 (Figure 2 and Table 3).

The coupling constants of H-3 at *δ*_H_ 4.08 (tt, *J* = 11.0, 5.0 Hz) indicated that the H-3 was axial, showing that the HO-3 was *β*-oriented. The chemical shift of H-25 (*δ*_H_ 2.20, sep, *J* = 6.8 Hz) and the ROESY correlation between H-26 and H-28 suggested that the double bond Δ^24(28)^ was *trans*-configuration, for H-25 resonated at significantly lower field (*δ*_H_ 2.63) in the *cis*-configuration (Figure 3) [14]. Therefore, compound **3** was identified as (24*E*)-5*α*,6*α*-epoxystigmasta-7,24(28)-dien-3*β*-ol.

## 3. Experimental 

### 3.1. General

Melting points were determined on a SGW X-4 melting point apparatus and were uncorrected. Optical rotations were measured on a JASCO P-1030 polarimeter. EI-MS, TOF-API-ES, ESI-MS and HR-TOF-ESI-MS spectra were acquired using a Q-Tof micro YA019 mass spectrometer. NMR experiments were performed on a Bruker AVANCE-600 spectrometer. HPLC purifications were carried out on a Waters 1525/2998 liquid chromatograph using SunFire Prep C18 column (250 × 10 mm, 5 μm). CC was performed on Sephadex LH-20 (Pharmacia) and YMC ODS-A (50 *μ*m). VLC was performed on silica gel (200-300 mesh, Yantai, China). Fractions were monitored by TLC (HSGF 254, Yantai, China) and spots were visualized by heating silica gel plates sprayed with 10% H_2_SO_4_ in H_2_O.

### 3.2. Animal Material

Specimen of *P. foliascens* was collected around Yongxing Island in the South China Sea in June 2007, and was identified by Prof. Li Jin-He (Institute of Oceanology, Chinese Academy of Sciences, China). A voucher sample (No. DS-PF01) was deposited in Laboratory of Marine Drugs, Department of Pharmacy, Changzheng Hospital, Second Military Medical University, China.

### 3.3. Extraction and Isolation

The fresh sponges (800 g, dry wt.) were extracted with acetone (1,500 mL, 3 times) at room temperature. The acetone extracts were concentrated under reduced pressure to give 55 g of a brown gum, which was partitioned between MeOH-H_2_O (9:1) and petroleum ether (PE) to afford 10 g of PE-soluble extract. The MeOH-H_2_O phase was diluted to 3:2 with H_2_O and extracted with CH_2_Cl_2_ to give 8 g of CH_2_Cl_2_-soluble extract. The PE-soluble extract was subjected to VLC on silica gel using CH_2_Cl_2_/MeOH (25:1, 10:1, 5:1 and 2:1) as eluent to afford twelve fractions (*Fr.* *A*−*Fr.* *L*). The *Fr.* *B* (200 mg) was subjected to CC repeatedly on Sephadex LH-20 and YMC ODS-A (50 *µ*m), and further purified by HPLC (88.7 % MeOH in H_2_O, 1.5 mL/min, detection 218 nm) to yield pure compounds **1** (1.6 mg, *t*_R_ = 65.1 min) and **2** (1.7 mg, *t*_R_ = 68.1 min). Similarly, the CH_2_Cl_2_-soluble extract was subjected to VLC on silica gel to give eight fractions (*Fr.* *M*−*Fr.* *T*). The *Fr.* *P* (130 mg) was subjected to CC on silica gel and HPLC (96 % MeOH in H_2_O, 1.5 mL/min, detection 254 nm) to yield compound **3** (2.5 mg, *t*_R_ = 27.0 min).

*20,24α-Dimethyl-12α-hydroxyscalaran-25,24-lactone* (*phyllofolactone M*, **1**): white powder (CHCl_3_), m.p. 237–239 °C; [*α*]^19^_D_ +61° (*c* 0.080, CHCl_3_); HR-TOF-ESI-MS: *m/z* 437.3033 (C_27_H_42_O_3_Na, calcd 437.3032); ^1^H- and ^13^C-NMR (C_5_D_5_N): see Table 1; ^13^C-NMR (150 MHz, CD_3_Cl): *δ* 8.6 (C-27), 16.6 (C-15), 16.9 (C-22), 16.8 (C-21), 18.1 (C-2), 18.3 (C-6), 18.4 (C-26), 21.7 (C-23), 24.0 (C-16), 24.4 (C-20), 24.3 (C-11), 28.5 (C-19), 36.1 (C-4), 36.6 (C-3), 37.0 (C-10), 37.6 (C-8), 40.0 (C-1), 40.3 (C-13), 41.8 (C-7), 49.9 (C-14), 52.3 (C-9), 58.4 (C-5), 70.1 (C-12), 78.6 (C-24), 133.4 (C-18), 165.2 (C-17), 172.6 (C-25).

*20,24β-Dimethyl-12α-hydroxyscalaran-25,24-lactone* (*phyllofolactone* B, **2**): white powder (CHCl_3_), m.p. 278–280 °C; [*α*]^19^_D_ +60° (*c* 0.085, CHCl_3_); ESI-MS: m/z 437.32 ([M*+*Na]^+^), 851.63 ([2M*+*Na]^+^); ^1^H-NMR (600 MHz, C_5_D_5_N): *δ* 6.04 (1H, br. s, HO-12), 5.09 (1H, br. s. H-12), 4.68 (1H, q, *J* = 6.7 Hz, H-24), 1.24 (3H, s, H-23), 1.20 (3H, d, *J* = 6.6 Hz, H-26), 0.87 (3H, s, H-22), 0.83 (3H, s, H-21), 0.78 (3H, s, H-19), 0.73 (3H, t, *J* = 7.2 Hz, H-27); ^13^C-NMR (150 MHz, C_5_D_5_N): *δ* 8.8 (C-27), 17.2 (C-15), 17.2 (C-22), 17.4 (C-21), 18.4 (C-2), 18.6 (C-26), 18.7 (C-6), 21.7 (C-23), 24.4 (C-16), 24.7 (C-20), 25.2 (C-11), 28.6 (C-19), 36.2 (C-4), 36.8 (C-3), 37.3 (C-10), 37.9 (C-8), 40.0 (C-1), 40.7 (C-13), 42.1 (C-7), 49.7 (C-14), 52.2 (C-9), 58.7 (C-5), 69.5 (C-12), 77.7 (C-24), 134.5 (C-18), 163.8 (C-17), 172.1 (C-25); ^13^C-NMR (150 MHz, CD_3_Cl): *δ* 8.6 (C-27), 16.8 (C-15), 16.8 (C-22), 16.8 (C-21), 18.1 (C-2), 18.3 (C-6), 18.5 (C-26), 21.9 (C-23), 24.1 (C-16), 24.4 (C-20), 24.1 (C-11), 28.5 (C-19), 36.1 (C-4), 36.6 (C-3), 37.0 (C-10), 37.6 (C-8), 40.0 (C-1), 40.2 (C-13), 41.8 (C-7), 49.6 (C-14), 52.2 (C-9), 58.5 (C-5), 70.1 (C-12), 78.2 (C-24), 133.6 (C-18), 165.1 (C-17), 172.7 (C-25).

*24(E)-5α,6α-Epoxystigmasta-7,24(28)-dien-3β-ol* (**3**): white powder (CHCl_3_), m.p. 245–247 °C; [*α*]^19^_D_ –6° (*c* 0.090, CHCl_3_); EI-MS: *m/z* 426, 408, 397, 393, 379, 269, 262, 251, 227, 218, 197, 175, 159, 149, 135, 121, 111, 109, 97, 95, 83, 81, 71, 69, 57, 55, 45, 43; TOF-API-MS: *m/z* 427 ([M*+*H]^+^); ^1^H- and ^13^C-NMR (CDCl_3_): see Table 3.

## 4. Conclusions

A new 20,24-bishomoscalarane sesterterpene, 20,24*α*-dimethyl-12*α*-hydroxy-scalaran-25,24-lactone (phyllofolactone M, **1**), and a new sterol, (24*E*)-5*α*,6*α*-epoxystigmasta-7,24(28)-dien-3*β*-ol (**3**), together with a known sesterterpene, phyllofolactone B (**2**), were isolated from the South China Sea sponge *P. foliascens* by chromatography methods. Phyllofolactone M (**1**) and phyllofolactone B (**2**) are epimers at C-24, and the previous NMR data assignment for phyllofolactone B (**2**) was revised on the basis of spectroscopic and physical data analysis.

## Figures and Tables

**Table 1:** ^1^H- (600 MHz) and ^13^C-NMR (150 MHz) data for **1** in C_5_D_5_N.

Position	^1^H (mult., *J* in Hz)	^13^C (mult.)	COSY	HMBC (H→C)	ROESY
1	*α*: 0.90 (m)	40.1 (t)	2*α*		
	*β*: 1.57 (m)		2*α*		11*α*
2	*α*: 1.45 (m)	18.5 (t)	1*β*, 2*α*, 3*α*		19
	*β*: 1.28 (m)				21
3	*α*: 0.81 (m)	37.0 (t)	2*α*		19
	*β*: 1.61 (m)			1, 5	
4	−	36.2 (s)			
5	0.89 (m)	58.8 (d)	6*β*		9, 19
6	*α*: 1.72 (m)	18.6 (t)	7*β*	5	
	*β*: 1.40 (m)		5		20, 21, 22
7	*α*: 0.88 (m)	42.1 (t)			9, 14
	*β*: 1.73 (m)		6*α*		15*α*
8	–	38.0 (s)			
9	1.81 (dd, 11.8, 2.4)	52.3 (d)	11*α*, 11*β*	11, 21, 22	5, 7*α*, 14
10	–	37.3 (s)			
11	*α*: 1.75 (m)	25.2 (t)	9, 12		1*β*
	*β*: 1.73 (m)		9, 12	9	21, 22, 23
12	5.05 (br. s)	69.5 (d)	11	9, 14	23
13	−	40.7 (s)			
14	1.88 (br. d, 12.3)	50.0 (d)	15*β*	8, 13, 15, 16, 22, 23	7*α*, 9, 16
15	*α*: 1.74 (m)	17.0 (t)	15*β*, 16	8	7*β*, 16
	*β*: 1.43 (m)		14, 15*α*, 16		16, 22, 23
16	2.15 (2H, m)	24.8 (t)	15*α*, 15*β*	14, 15, 17, 18	14, 15*α*, 15*β*, 24, 26
17	−	163.8 (s)			
18	−	134.2 (s)			
19	0.79 (s)	28.7 (q)		3, 4, 5, 20	2*α*, 3*α*, 5
20	a: 1.52 (m)	24.8 (t)	27	4, 19, 27	6*β*, 21
	b: 1.14 (m)		27	3, 27	
21	0.82 (s)	17.4 (q)		1, 5, 9, 10	6*β*, 11*β*, 20
22	0.86 (s)	17.2 (q)		7, 8, 9, 14	6*β*, 11*β*, 15*β*, 23
23	1.24 (s)	21.5 (q)		12, 13, 14, 18	11*β*, 12, 15*β*, 22
24	4.75 (q, 6.7)	78.2 (d)	26	17, 18, 25, 26	16
25	−	172.0 (s)			
26	1.19 (d, 6.7)	18.5 (q)	24	17, 24	16
27	0.74 (t, 7.4)	8.9 (q)	20	4, 20	
HO-12	5.92 (br. s)				

**Table 2:** Comparison of ^13^C-NMR and Physical Data for Compounds **1**, **2**, and reported phyllofolactone B.

Compound	C-24 in C_5_D_5_N	C-24 in CD_3_Cl	M.P. (°C)	Optical Rotation (CHCl_3_)
**1**	78.2	78.6	237–239	[*α*]^19^_D_ = +61°
2	77.7	78.2	278–280	[*α*]^19^_D_ = +60°
phyllofolactone B		78.6	232–234	[*α*]^20^_D_ = +61.9°

**Table 3:** ^1^H- (600 MHz) and ^13^C-NMR (150 MH) data for **3** in CD_3_Cl.

Position	^1^H (mult., *J* in Hz)	^13^C (mult.)	^1^H-^1^H COSY	HMBC (H→C)	ROESY
1	*α*: 1.55 (m)	33.0 (t)	2*β*		9, 11*α*
	*β*: 1.61 (m)		2*β*	9	
2	*α*: 1.87 (m)	30.9 (t)	2*β*, 3		
	*β*: 1.45 (m)		1*α*, 1*β*, 3		19
3	4.08 (tt, 11.0, 5.0)	67.7 (d)	2*α*, 2*β*, 4*α*, 4*β*		4*α*
4	*α*: 1.78 (dd, 13.0, 3.5)	39.3 (t)	3, 4*β*	2, 3, 5, 10	3
	*β*: 2.14 (t, 12.0)		3, 4*α*	3	19
5	−	76.0 (s)			
6	3.63 (br. d, 4.6)	73.7 (d)		5, 7, 8, 10	7
7	5.36 (m)	117.6 (d)	6	5, 9, 14	6, 15*α*, 15*β*
8	−	144.0 (s)	7		
9	1.96 (m)	43.5 (d)	11*β*		1*α*, 12*α*
10	−	37.1 (s)			
11	*α*: 1.57 (m)	23.0 (t)	11*β*, 12*α*		1*α*
	*β*: 1.31 (m)		9, 11*α*, 12*α*, 12*β*		19
12	*α*: 1.32 (m)	39.5 (t)	11*α*, 11*β*, 12*β*		9
	*β*: 2.09 (m)		11*β*, 12*α*	9	21
13	−	43.9 (s)			
14	1.91 (m)	54.7 (d)	15*α*, 15*β*	9, 13	16*α*
15	*α*: 1.60 (m)	22.1 (t)	14, 15*β*		
	*β*: 1.49 (m)		14, 15*α*, 16*α*		18
16	*α*: 1.59 (m)	27.8 (t)	15*β*, 16*β*	18	14
	*β*: 1.94 (m)		15*β*, 16*α*		18
17	1.32 (m)	55.9 (d)	20	18	
18	0.60 (s)	12.1 (q)		12, 13, 14, 17	15*β*, 16*β*, 20, 21, 29
19	1.09 (s)	18.8 (q)		1, 5, 9, 10	2*β*, 4*β*, 11*β*, 18
20	1.40 (m)	36.8 (d)	17, 21, 22b		18
21	1.01 (d, 6.5)	18.8 (q)	20	17, 20, 22	12*β*, 18
22	a: 1.42 (m)	35.1 (t)	22b, 23a, 23b		
	b: 1.10 (m)		21, 22a, 23a, 23b		
23	a: 2.08 (m)	25.8 (t)	22a, 22b, 23b	22, 24, 25, 28	
	b: 1.87 (m)		22a, 22b, 23a	22, 24, 25, 28	
24	−	146.9 (s)			
25	2.20 (sep, 6.8)	34.8 (d)	26, 27	23, 24, 26, 27, 28	
26	0.99 (d, 6.8)	22.2 (q)	25	24, 25, 27	28
27	0.98 (d, 6.8)	22.1 (q)	25	24, 25, 26	
28	5.19 (q, 6.8)	115.7 (d)	29	22, 23, 24, 25, 29	26
29	1.58 (d, 6.8)	13.2 (q)	28	24, 28	18
